# Interaction of Coenzyme Q10 with Liposomes and its Impact on Suppression of Selenite – Induced Experimental Cataract

**DOI:** 10.15171/apb.2018.001

**Published:** 2018-03-18

**Authors:** Medhat Wahba Shafaa, Amany Hasan Elshazly, Amira Zaki Dakrory, Maha Reda Elsyed

**Affiliations:** ^1^Physics Department, Medical Biophysics Division, Faculty of Sciences, Helwan University, Cairo, Egypt.; ^2^Departments of Biochemistry and Pharmacology, Research Institute of Ophthalmology, Giza, Egypt.; ^3^Physics Department, Faculty of Women for arts, Science and Education Ain Shams University, Cairo, Egypt.

**Keywords:** Liposomes, Coenzyme Q10, DSC, FTIR, Cataract, Lens Soluble Proteins

## Abstract

***Purpose:*** To stress the influence of Coenzyme Q10 (CoQ10) on the structural properties of liposomes as model membranes and to investigate the possible role of CoQ10 or CoQ10 doped in liposomes when topically instilled as eye drops, in preventing cataract.

***Methods:*** The molecular interaction between liposomes and Coenzyme Q10 was examined using differential scanning calorimetry (DSC) and Fourier transform infrared spectroscopy (FTIR). Rat pups were randomly divided into six groups comprising 15 pups. Group (1), control group. Group (2), untreated model of cataract, received a single subcutaneous injection of sodium selenite. Instillation of pure CoQ10 (Group 3), CoQ10 encapsulated into neutral (Group 4), positive (Group 5) and negative (Group 6) Dipalmitoyl phosphatidylcholine (DPPC) liposomes on the opacification of lenses in rat pups after sodium selenite injection was topically received.

***Results:*** The incorporated CoQ10 is probably associated with lipid bilayers where it interacts to a large extent and perturbs them. This results in strong broadening and shift to lower temperature (94°C) of the major characteristic endothermic peak of pure DPPC at 105°C. FTIR showed that the incorporation of CoQ10 into DPPC induces a conformational change in the polar region of DPPC. Ophthalmological and Biochemical studies revealed that CoQ10 alone followed by negatively charged liposomes doped with CoQ10 are more effective in reducing the progress of cataract as well as improving the lens soluble proteins levels and total antioxidant capacity.

***Conclusion:*** The interactions of CoQ10 with membrane systems may contribute to a better understanding of CoQ10 physiological properties and the development of therapeutically advanced systems.

## Introduction

Phospholipids, such as phosphatidylcholine, are major targets that are subject to the damage caused by free radicals in cellular membranes. Lipid oxidation that causes cellular damage is strongly associated with ageing, carcinogenesis and other diseases.^1^ Free radicals (which are molecules or atoms with unpaired electrons) are passivated by reducing agents. Therefore, they are defined as antioxidants as they limit the oxidative damage to biological structures. Imbalance between pro-oxidant and antioxidant agents is termed oxidative stress, which in may result in oxidative damage. The state oxidative stress results in the elevation of free radicals which can react with cellular lipids, proteins, and nucleic acids leading to local injury and eventual organ dysfunction. Lipids are, probably, the most susceptible bio-molecule to be attacked by free radicals.

Fortunately, a number of defense protective mechanisms known as the “antioxidant defense system” are developed in our bodies. These are enzymes and non-enzymatic antioxidants like vitamins (classified to water soluble and lipid soluble vitamins) and minerals.^2^

Cataract is a white, opaque lesion that forms on the normally transparent lens of the eye. It occurs as a result of damage to the protein structure of the lens. In recent years it has been identified that oxidative stress (due to free radicals) is a major contributing factor to the development of age-related (senile) cataract and that the normal antioxidant protective mechanisms in the lens have been found to be significantly deteriorated in cases where senile cataract occurs.

A significant body of epidemiological evidence has been published regarding the potential role of antioxidants in the prevention of cataract. Although the majority of epidemiological studies have shown a positive correlation between higher dietary antioxidant intake and decreased cataract formation, conflicting conclusions exist. There are some studies indicating that the disturbance in the oxidative state of the lens can be corrected by giving various antioxidants.^3^

The only endogenously synthesized antioxidant existing in all cell membranes of our body is Coenzyme Q10 (CoQ10). It is essential for the production of adenosine triphosphate (ATP), which is the energy source for all living cells. Many cardiovascular and neurodegenerative disorders has been proposed to be treated or possibly even prevented by the help of CoQ10. Therefore, it has become one of the most popular nutritional supplements. Membrane phospholipids are efficiently protected from peroxidation and also mitochondrial DNA and membrane proteins from free-radical-induced oxidative damage by CoQ10.^4^ The excellent ability to scavenge free radicals makes CoQ10 attractive to be examined as a potential anti-cataract agent for the first time. However, instability to light and extreme lipophilicity of CoQ10 are hampering its bioavailability as a therapeutic agent.

Because of its high molecular weight and poor water solubility, CoQ10 has very low oral bioavailability from the gastrointestinal tract.^5^ Several formulations have been adopted to improve in vitro dissolution and absorbability of CoQ10, among which are liposomes.^6^

Liposomes are considered acceptable and superior drug delivery systems because they are biocompatible, biodegradable and nontoxic.^7^ Liposomes are useful tools to investigate the significance of the antioxidant-membrane interactions for antioxidant activity due to the resemblance between the liposomes and membrane bilayer core. With respect to treating oxidant induced tissue injuries, it has been demonstrated that encapsulation of antioxidants in liposomes promotes their therapeutic potential against oxidant-induced tissue injuries, presumably by liposomes facilitating the intracellular uptake and extending the half-lives of the encapsulated antioxidants.

The main aim of this work is to investigate the impact of CoQ10, as an antioxidant, on the structural properties of model lipid membranes and to estimate the subtle perturbation of the lipid bilayer structure using two non- invasive techniques such as differential scanning calorimetry (DSC) and Fourier transform infrared spectroscopy (FT-IR). The work also investigates the possible role of CoQ10 or CoQ10 doped in liposomes when topically instilled as eye drops, in preventing selenite – induced experimental cataract on the basis of biophysical / biochemical monitoring of CoQ10 behavior.

## Materials and Methods

L-a-Dipalmitoyl phosphatidylcholine (DPPC) in powder form and of purity 99% of molecular weight of 734 was used in this work. DPPC, Dicetyl phosphate (DCP), molecular weight of 546.9 of purity 99% and, Stearyl amine (SA), molecular weight of 269.5 of purity 99% were all purchased from Sigma (ST. Louis, Mo, USA). Trizma buffer, molecular weight of 121.1, Coenzyme Q10 (CoQ10), molecular weight of 863.358 were purchased from EIPICO, Egypt. All other reagents and solvents used in this work were of research grade.

### Preparation of Coenzyme Q10 - doped liposomes

DPPC: CoQ10 molar ratio 7:2 was used to prepare neutral liposomal multilamellar vesicles (MLVs) using the method of Deamer and Uster.^8^ In 50 ml round bottom flask, 10 mg of DPPC and 3.4 mg of the drug powder at molar ratio 7:2 were transferred. Then 10 ml of ethanol (EtOH) was added, and the flask was shaken until all lipids dissolve in the EtOH. The solvent was then evaporated under vacuum using rotary evaporator until a thin dry film of lipid was formed. 10 ml of buffer (10 mM Trizma at pH = 7) was then added to the flask which was flashed through with nitrogen stream and immediately stoppered. The flask was mechanically shaken for 1 h at temperature above the phase transition temperature of lipids (45°C). The suspension was then centrifuged at 8000 rpm for 20 min and the supernatant was discarded. The liposomes were then re-suspended in 10 ml buffer solution. Either 0.506 mg of SA or 1.027 mg of DCP was added to the lipid composition to introduce a net positive or negative charge, respectively. Control liposomes were prepared following the same classical methods as mentioned using only 10 mg of DPPC.

### Encapsulation efficiency measurements

The entrapment efficiency (EE) of CoQ10 incorporated into liposomes was determined using a spectrophotometer (Uvikon 930, Italy). The wavelength was adjusted at the resonance absorption peak of CoQ10 which is located at 275 nm. The absorption of the supernatant of each sample was compared with the standard curve that relates absorption to the concentration of CoQ10. The encapsulation efficiency was found to increase more than 90% when mixing CoQ10 with the lipid powder before dissolving it in ethanol. Entrapment Efficiency (EE) was calculated as follows:

EE = [Total drug input (mg) – drug in supernatant (mg)] × 100 */* [Total drug input (mg)].

### DSC measurements

Differential scanning calorimetry (DSC) experiments were carried out using TA-50 WSI (Schimadzu, Japan) calibrated with indium to investigate the thermal behavior of lyophilized samples of empty and CoQ10-loaded multilamellar liposomes. Analysis was performed on 5-mg samples sealed in standard aluminum pans. The thermogram of each sample covers the 25-200°C temperature range at a scanning rate of 5°C/min.

### FTIR Spectroscopy

FTIR spectra of lyophilized samples of DPPC liposomes and DPPC liposomes encapsulated CoQ10 deposited in KBr disks were recorded on a NICOLET 6700 FTIR spectrometer (Thermo Scientific, Cambridge, England). Scanning was carried out at room temperature, in the range 400–4,000 cm^-1^ at a speed of 2 mm/s and a resolution of 4 cm^-1^.

### Experimental Animals

Ninety male and female rat pups aged 10 days old were used, with a weight range of 20-25 grams. The rats were divided into six groups, each group composed of 15 rats. They are obtained from the animal house of The Research Institute of Ophthalmology, Giza, Egypt. Suckling rats were housed with their parents in separate cages, and parents were given food and water. The animal room was well ventilated with a regular 12:12- hour light/dark cycle maintained throughout the experimental period. The experiment was performed in accordance with the ARVO rules for use of animals in ophthalmic and vision research.

### Groups and Cataract Induction

Rat pups were randomly divided into six groups comprising 15 pups each; Group (1), control group, received saline eye drops twice daily. Group (2), untreated model of cataract, received a single subcutaneous injection of sodium selenite (19 µmol/kg body weight) on the 10^th^ postpartum day. Animals in this group received one drop of the vehicle solution, twice daily. Vehicle eye drops were prepared without adding COQ10. Group (3), pups were injected with Na selenite and received one drop of COQ10 eye drops, twice a day. COQ10 eye drops (10 mg) were prepared in 30 ml of 20% dilute ethyl alcohol,^9^ Group (4), pups were injected with Na selenite and received one drop of DPPC encapsulated with neutral COQ10 eye drops, twice a day. Group (5), pups were injected with Na selenite and received one drop of DPPC encapsulated with positive COQ10 eye drops, twice a day. Group (6), pups were injected with Na selenite and received one drop of DPPC encapsulated with negative COQ10 eye drops, twice a day. After 13 days of Selenite injection and treatment, rats were sacrificed.

### Ophthalmic Examination

When the pups first opened their eyes (approximately 16 days afterbirth), a slit-lamp biomicroscopic examination was performed on each eye of each rat pup to provide a morphological evaluation of any lenticular opacification. Prior to performing the examination, mydriasis was achieved by tropicamide eye drops 1% (Alcon Egypt). Direct ophthalmoscope (Wech Allen) and hand held slit lamp (Carl Zeiss) were used to detect the presence of cataract and other anterior chamber changes. Rats with anterior segment changes were excluded. The degree of cataract was graded as follow: grad+ (faint lens opacity, just detectable), grade++ (dense opacity with hazy view of the fundus, slightly obstructed red reflex), and the last grade**+++** (dense lens opacity with totally obstructed red reflex).^10^ Photography was done on day 7 and on day 13 to detect the progress of cataract using Topcon fundus camera with anterior segment facility.

At the end of the experimental period (postpartum day 23), animals were anaesthetized using diethyl ether, and lenses were dissected out by the posterior approach. Lenses were immediately blot dried on a blotting paper and weighed and kept in clean glass vials at -20°C till analyzed.

### Preparation of lenses for analysis

Lenses from each group were homogenized in ten times their mass of 50 mM phosphate buffer (pH 7.2), and centrifuged at 12,000 rpm for 15 min at 4°C. The supernatant was stored at - 70°C in aliquots until used for the analysis.

### Protein assay measurements

Lens proteins were assayed using the method described by Lowry et al^11^ by using bovine serum albumin as a standard.

### Analysis of total antioxidant capacity 

Total antioxidant capacity was measured using commercial kits obtained from Biodiagnostic (Egypt). Absorbance was read at 590 nm against blank on a spectrophotometer.

## Results

### DSC Studies

**Figure 1:**
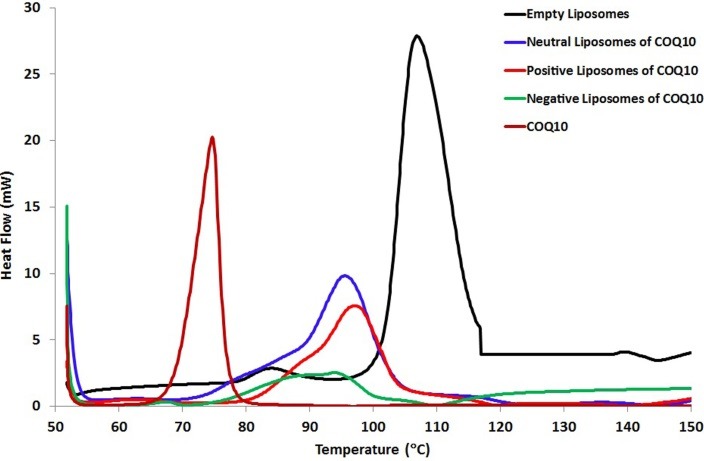
DSC diagrams of liposomes made of pure DPPC, liposomes doped with CoQ10 and pure CoQ10.

Pure DPPC vesicles upon dehydration when submitted to DSC analysis, showed a major endothermic peak at 105°C (Figure 1), in accordance with previous studies.^12,13^ The pre-transition temperature was around 80°C for empty DPPC liposomes. A sharp endothermic peak at about 75°C was observed for CoQ10 alone.

The incorporation of CoQ10 into neutrally or positively charged liposomes resulted in noticeable broadening and shift to lower temperatures 96°C and 98°C respectively in comparison to the main endothermic peak of empty DPPC that exists at 105°C.

The pre-transition temperature peak for neutral and positive DPPC/ CoQ10 liposomes disappeared, which indicates that CoQ10 interacts with the polar head groups of phospholipids. The disappearance of the pre-transition was sensitive for the incorporation of biomolecules into lipid bilayer.

Upon the incorporation into negatively charged liposomes, the pronounced effect of CoQ10 was observed in comparison with the other liposome formulations. The incorporated CoQ10 was probably associated with the lipid bilayers, interacted to a large extent with them, and perturbed them which resulted in the strong broadening and shift to lower temperature 94°C of the major characteristic endothermic peak of empty DPPC at 105°C. The incorporation of CoQ10 resulted in a very high miscibility of CoQ10 with phospholipid bilayer which results in the disappearance of its endothermic peak at about 75°C (Figure 1).

### FTIR Studies

**Figure 2:**
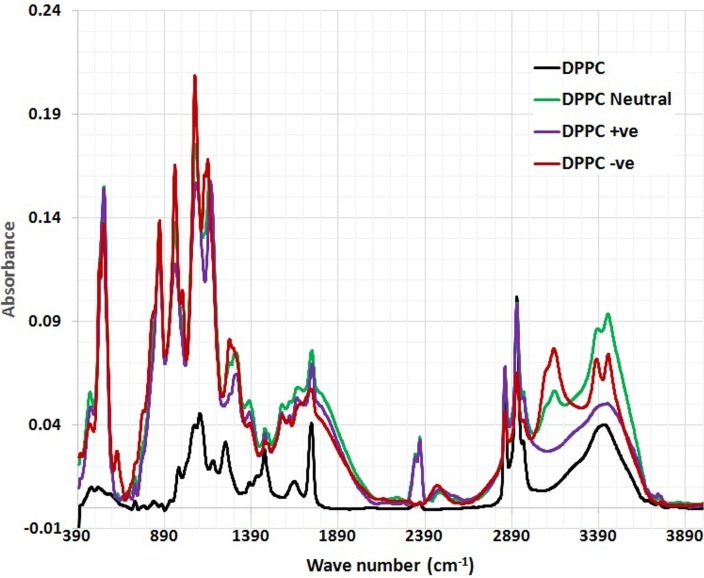
The full FTIR spectra of DPPC and DPPC/ CoQ10 liposomal samples.

FTIR spectra of empty lyophilized DPPC liposomes compared with CoQ10/DPPC liposomal samples in the region of 4000–400 cm^–1^ are presented in (Figure 2)**.** The spectrum of the DPPC liposomes displays the main characteristic bands, especially those due to the symmetric and antisymmetric PO_2_- stretching vibrations at 1090 and 1220 cm^-1^, respectively, the CH_2_ bending vibration CH_2_ near 1470 cm^-1^, the carbonyl stretching vibration C=O at 1734 cm^-1^, the OH stretching and bending vibrations at 3470 and 1640 cm^-1^, respectively and The symmetric and antisymmetric stretching vibrations of the CH_2_ in the acyl chain around 2850 and 2920 cm^-1^, respectively. These findings were in good accordance with the data reported in the literature.^14^

**Figure 3:**
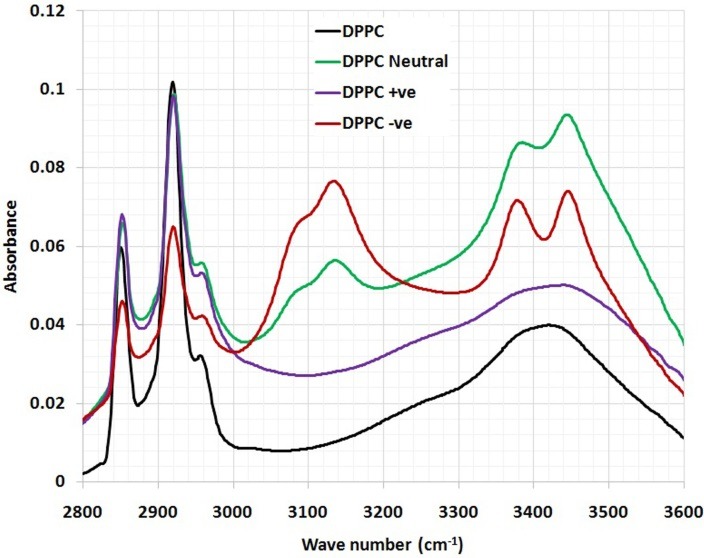
The magnified part (3500-2800 cm^-1^) of FTIR spectra of DPPC and DPPC/ CoQ10 liposomal samples.

**Figure 4:**
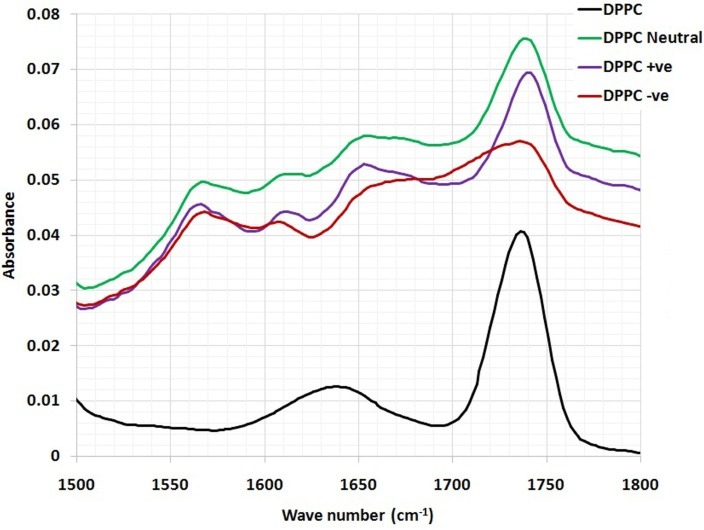
The magnified part (1800-1500 cm^-1^) of FTIR spectra of DPPC and DPPC/ CoQ10 liposomal samples.

**Figure 5:**
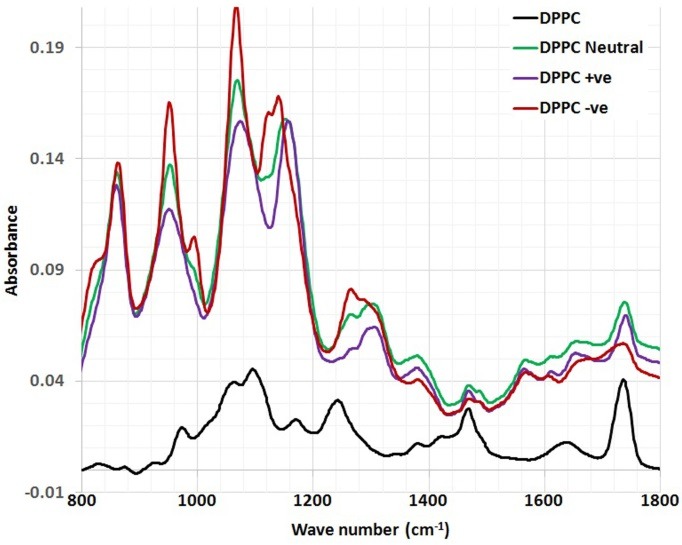
The magnified part (1800-800 cm^-1^) of FTIR spectra of DPPC and DPPC/ CoQ10 liposomal samples.

The detailed spectral analyses were performed in three distinct wave number regions, namely 3500–2800 cm^–1^ (Figure 3), 1800–1500 cm^–1^ (Figure 4) and 1800–800 cm^–1^ (Figure 5), since identifiable Raman bands are observed mainly in these regions only.

Incorporation of CoQ10 into neutrally, positively and negatively charged DPPC liposomes resulted in significant change in the frequency of the antisymmetric CH2 stretching bands in the acyl chain observed in (Figure 3).

In order to examine the interaction of CoQ10 with the glycerol backbone near the head group of phospholipids in the interfacial region, the C=O stretching band was analyzed. The wavenumber variation of this band is shown in (Figure 4). As seen from (Figure 4), the wavenumber value of C=O group was shifted to higher values (from 1737cm^-1^ to 1739.5 and 1737.5 cm^-1^) for the neutral and negatively charged liposome samples containing CoQ10, respectively, without any evidence of hydrogen bonding formation. The absorption bands of ester C=O were sensitive to changes in the polarity of their local environments and were influenced by hydrogen bonding and other interactions**.** Therefore, changes in the contours of the ester C=O absorption band can often be interpreted in terms of structural and/or hydration changes of the bilayer polar/apolar interface**.**^15^ The wavenumber value of C=O group exhibits a shift towards higher frequency (from 1737 cm^-1^ to 1741.4 cm^-1^) for the positive liposomes sample containing CoQ10, implying dehydration about these functional groups in the interfacial region of the lipid membranes. Therefore, any change in the spectra in this region can be attributed to an interaction between CoQ10 and the polar/apolar interfacial region of the membrane.

The interesting finding that can be concluded from (Figure 5) is that the CH_2_ bending vibration mode which is located at 1468 cm^-1^ is affected by the incorporation of CoQ10 into DPPC liposomal preparation. As can be depicted from the (Figure 5), the wavenumber is shifted towards higher frequency (1469.515 cm^-1^) after the incorporation of CoQ10 into all formulations of DPPC liposomes.

The interaction between CoQ10 and the head group of DPPC liposomes was observed by PO_2_- antisymmetric stretching band, which was located at 1242 cm^-1^. Figure 5 shows the PO_2_- antisymmetric stretching band for DPPC liposomes in the absence and presence of CoQ10. As can be shown from (Figure 5), the wavenumber was shifted towards higher frequency 1265 cm^-1^ after the incorporation of CoQ10 into neutrally, positively and negatively charged DPPC liposomes, respectively.

An interesting peak appeared corresponding to the aliphatic phosphate stretching at 995 cm^-1^ upon the encapsulation of CoQ10 into negatively and positively charged DPPC liposomes, respectively. This was possibly due to the immobilizing effect of CoQ10 to the phosphate group moiety, (Figure 5). Table 1 summarizes the chemical shifts observed for CoQ10 after the incorporation into different formulations of DPPC liposomes.

**Table 1:** The chemical shifts observed for CoQ10 after the incorporation into different formulations' of DPPC liposomes.

**Peak assignment**	**Wave number (cm**^-1^**)**	**DPPC (Control)**	**DPPC neutral**	**DPPC +ve**	**DPPC** **-ve**
Symmetric stretching vibrations of the CH_2_ in the acyl chain	2800-2855	2850	2852.247	2852.247	2852.247
Anti-Symmetric stretching vibrations of the CH_2_ in the acyl chain	2920-3000	2920	2921.673	2919.745	2919.745
OH stretching vibrations	3400-3470	3419	3444.296	3438.51	3446.224
OH bending vibrations	1640-1645	1640	1652.722	1652.722	1652.722
Carbonyl stretching vibrations C=O	1730-1740	1737	1739.504	1741.433	1737.576
CH_2_ bending vibrations	1456-1470	1468	1469.515	1469.515	1469.515
Aliphatic phosphates (P-O-C stretch)	990-1050	-------	-------	995.1046	995.1046
Anti-Symmetric PO_2_ stretching vibrations	1215-1260	1242	1265	1265	1265

### Ophthalmic studies

**Figure 6:**
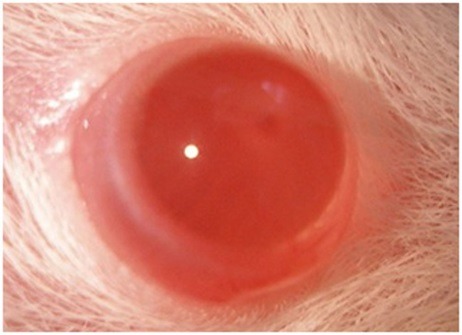
Anterior segment photography showing clear lens (group 1: normal).

**Figure 7:**
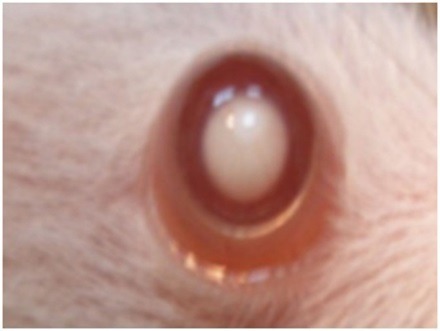
Anterior segment photography showing; complete cataract (grade+++)(group 2: untreated).

**Figure 8:**
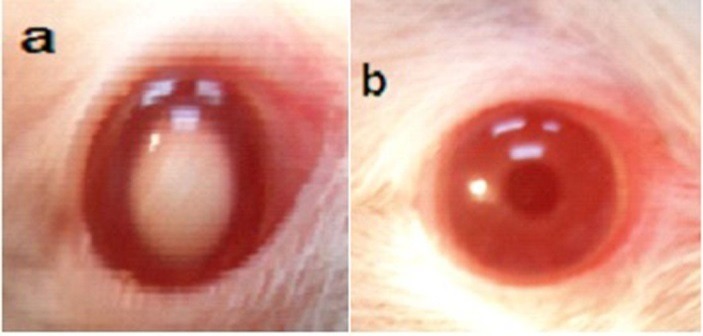
Anterior segment photography showing; a) nuclear cataract, grade+, b) clear lens (group 3: treated with free CoQ10).

**Figure 9:**
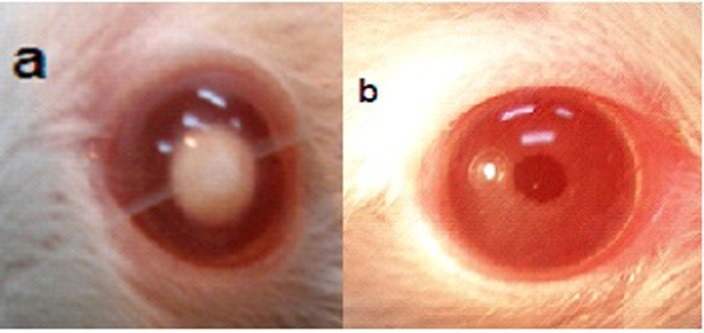
Anterior segment photography showing; a) nuclear cataract of grade++, b) clear lenses (group 4: treated with CoQ10 doped in neutral liposomes).

**Figure 10:**
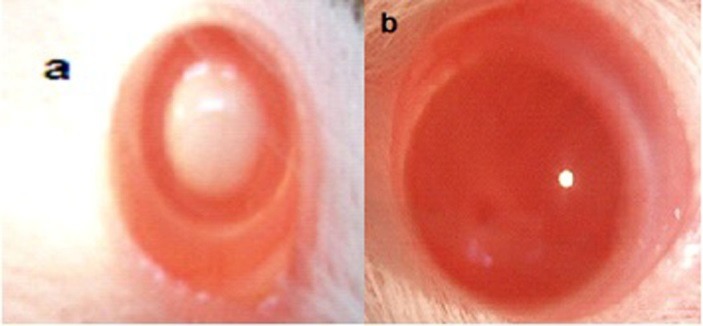
Anterior segment photography showing; a) nuclear cataract of grade+++, b) clear lenses (group 5: treated with CoQ10 doped in positive liposomes).

**Figure 11:**
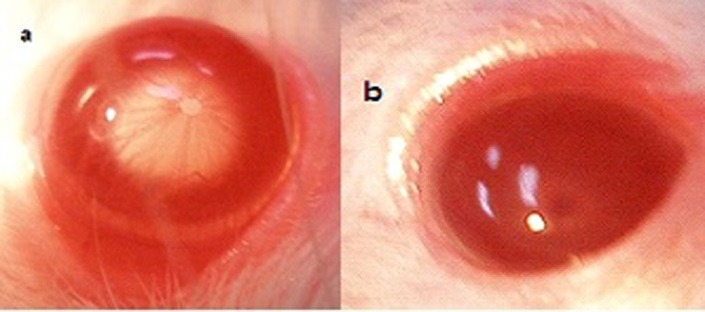
Anterior segment photography showing; a) faint nuclear cataract of grade+, b) clear lenses (group 6: treated with CoQ10 doped in negative liposomes).

Ophthalmic examination showed that 100% of rats' lenses (10 rats) in group1 (normal) remained clear with no visible opacification as shown in (Figure 6)**.** In group 2 (untreated cataract model in which rats were given Na selenite alone), 100 % of lenses showed nuclear cataract of grade+ or grade++. The opacity started on the third day after injection of Na selenite and increased rapidly in intensity on the following days extending to dense white nuclear cataract of grade+++ (Figure 7). In group 3, model cataract was induced and Coenzyme Q10 was instilled topically and daily for 13 days. Only 10% of rats showed faint nuclear opacification of grade+ (Figure 8a) which starts on day seven after Na selenite injection with no detectable increase in intensity on the following days. The lenses of remaining rats (90%) are clear as shown in (Figure 8b). On the other hand, in groups 4, 5, 6 model cataract was induced and Coenzyme Q10 encapsulated with neutral, positive, and negative DPPC liposomes drops were daily instilled respectively for 13 days. Ophthalmic examination reveals that about 20 % of rats in group 4 showed dense nuclear cataract of grade++. The lenses of remaining rats (80%) are free (Figure 9 a, b). In group 5**,** about 40% of rats' lenses showed nuclear opacification of grade +++. The lenses of remaining rats (60%) remain clear (Figure 10 a, b)**.** Whereas, in group 6 only 10% of rats have nuclear cataract of grade+ and the lenses of remaining rats (90%) remain clear (Figure 11 a, b).

Figure 12 summarizes the effect of instillation of CoQ10, CoQ10 encapsulated into neutral, positive and negative DPPC liposomes on the opacification of lenses in rat pups after sodium selenite injection.

**Figure 122:**
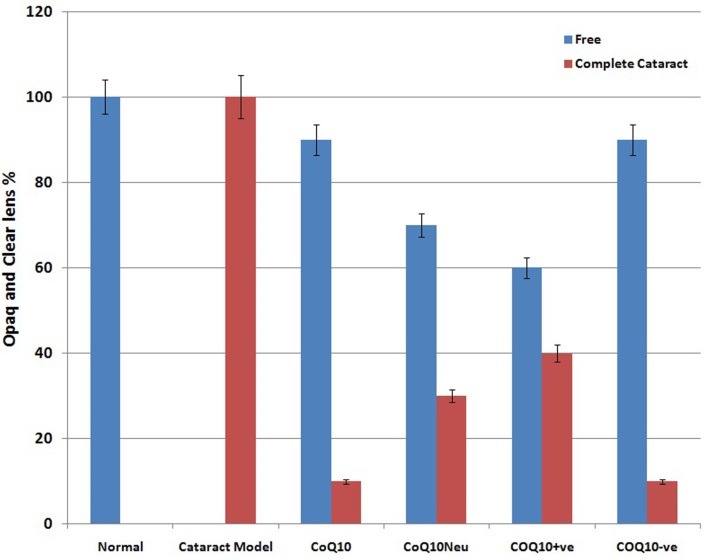
Comparison between complete cataract and clear lenses % between all groups.

The mean levels of soluble proteins and total antioxidant capacity (at the 13^th^ day of experiment) are shown in Table 2**.** Control rats (group1) showed normal values of the estimated parameters. Regarding induction of cataract model in group2, rats' lenses exhibited noticeable deterioration in the tested parameters. The mean levels of soluble proteins and total antioxidant capacity were reduced significantly from normal to 2±0.632 µg/g wet wt. and 0.096±0.0030 mmol /gm lens respectively as in Table 2**.**

As for the treated model rats, topical instillation of the tested drugs to groups 3,4,5,6 result in a highly significant improvement of both the soluble proteins and total antioxidant capacity as compared to the untreated model rats as well as normal rats. Soluble proteins mean values reach 40±0.632, 14±0.894, 10±1.414 and 17±0.408 µg/g wet wt. respectively. While total antioxidant capacity mean values are 0.41±0.0105, 0.289±0.0090, 0.143±0.0058, and 0.296±0.0045 mmol/gm lens respectively Table 2.

It can be noticed that the highest levels were in pure COQ10 eye drops followed by negative then neutral liposomes while positively charged liposome shows the lowest level. That runs in accordance to previous studies that observed the efficacy of pure COQ10 in preventing the effects of light-induced oxidative stress and apoptosis in human lens epithelial cells in a cell culture model.^16^ COQ10 has an antioxidant activity against oxygen species which is responsible for the oxidative stress.^17^ Coenzyme Q10 exhibited a markedly anti-cataract effect with the percentage of lens opacity being about 53% at the final examination. The mean activities of superoxide dismutase and reduced glutathione are significantly higher in the Coenzyme Q10-treated group than in the cataract model group, while malondialdehyde is significantly lower.^18^

The present results that favor the negatively charged loaded liposomes are similar to those reported by Ogiso et al^19^ who clarified the effects of the surface charges of liposomes on percutaneous absorption. They proved that both in vitro penetration rate and in vivo percutaneous absorption of drugs entrapped in negatively charged liposomes are significantly higher than that of positively charged ones.

**Table 2:** Mean levels of total antioxidant and soluble protein in lenses of rat pups studied groups.

**Groups** **Parameters**	**Group 1 normal**	**Group 2 untreated model of cataract**	**Group 3** **coQ10**	**Group 4** **neutral**	**Group 5** **+ve**	**Group 6** **-ve**
**Mean level of soluble proteins** (µg/g wet wt)±SD	7±1.41	2±0.63	40±0.63	14±0.89	10±1.41	17±0.40
**P1**		0.0312^*^	0.0021^*^	0.0021^*^	0.0108^*^	0.0021^*^
**P2**			0.0029^*^	0.0025^*^	0.0027^*^	0.0021^*^
**Total antioxidant capacity** (mmol /g lens)	0.209 ±0.0047	0.096 ±0.0030	0.416 ±0.0105	0.289 ±0.0090	0.143 ±0.0058	0.296 ±0.0045
**P1**		0.0010^*^	0.0023^*^	0.0010^*^	0.0023^*^	0.0017^*^
**P2**			0.0011^*^	0.0011^*^	0.0021^*^	0.0011^*^

Data are expressed as mean value ± SD. N=10 P^*^ is considered significant if < 0.05 P1 compared to normal (group1). P2 compared to model of untreated cataract (group2).

## Discussion

DSC is a fundamental technique for the characterization of membrane behavior, providing all thermodynamic parameters for temperature-induced transitions. The temperature at which a transition from the gel phase to the rippled phase takes place is called the pre-transition temperature and it is mainly related to the polar region of phospholipids. Subsequently, the melting of bilayer from the rippled phase to the liquid phase occurs at the main transition temperature (T_m_). The melting point (T_m_) represents the peak temperature of the endotherm for the lipid gel-to fluid phase transition recorded during the heating scan.

The DPPC vesicles are used as model membranes since this phospholipid is able to mimic many aspects of biological membranes. The presence of a compound in the DPPC membranes can influence the thermotropic parameters of the vesicle transition. The incorporation of CoQ10 into neutrally or positively charged liposomes results in noticeable broadening and shift to lower temperatures. This suggests that CoQ10 has a significant effect on the acyl chains of DPPC bilayers creating a conformational disorder within the acyl chains of phospholipids and decreasing the transition cooperatively of lipid acyl chains. These findings are in good accordance with the data reported in the literature.^20,21^

The change in phase transition suggests that the incorporated CoQ10 can be localized near the interface region and within the hydrophobic core thus giving rise to CoQ10 enriched microdomains.

Protein and DNA structure, hydration, and binding of biomolecules have been studied using vibrational spectroscopy, as a combined theoretical and experimental approach. FTIR spectroscopy is therefore used to monitor changes in the liposomal membrane structure by analyzing the frequency of different vibrational modes representing the acyl chains, interfacial, and head group region of lipid molecules.

Incorporation of CoQ10 into neutrally, positively and negatively charged DPPC liposomes results in significant change in the frequency of the antisymmetric CH_2_ stretching bands in the acyl chain observed in (Figure 3) implying that CoQ10 creates a conformational disorder within the acyl chains of phospholipids. In other words, it has significant effect on the order of the membrane.

The interaction of CoQ10 with the glycerol backbone near the head group of phospholipids in the interfacial region, the C=O stretching band is analyzed. CoQ10 tends to reduce the formation of hydrogen bonding in the interfacial region of DPPC liposome, implying the existence of free carbonyl groups in the system. Some H_2_O molecules are probably replaced by CoQ10 from the interfacial region leading to an increase in the number of free carbonyl groups.

The CH_2_ bending vibration mode which is located at 1468 cm^-1^ is affected by the incorporation of CoQ10 into DPPC liposomal preparation. This reveals the presence of disordering effect in acyl chain packing in the gel phases of phospholipids, in accordance with DSC studies. This may assume that the molecules of CoQ10 act as small spacers of the polar head group, leading to a slight disorder in the hydrocarbon chains.

The PO_2_- antisymmetric stretching band for DPPC liposomes in the absence and presence of CoQ10. As can be shown from (Figure 5), the wavenumber is shifted towards higher frequency 1265 cm^-1^ after the incorporation of CoQ10 into neutrally, positively and negatively charged DPPC liposomes. This implies the reduction of hydrogen bonding between the liposome head group and CoQ10 indicating an increase in the dehydration of the phosphate group. In accordance with the empirical rules, decreasing frequency values indicate an increase in the strengthening of existing hydrogen bonding or in the formation of new hydrogen bonding between the components.^22^

Ophthalmological and biochemical examination results run in parallel with the biophysical results of the present work. The presented DSC scans show that the incorporation of CoQ10 into negatively charged liposomes may cause a distinct broadening of the main endothermic peak when compared with other liposome formulations.

Moreover, FTIR spectroscopy reveals that incorporation of CoQ10 into DPPC liposomes shows significant conformational changes in the phospholipids structure. This can be attributed to an interaction between CoQ10 and the polar/apolar interfacial region of the membrane. Those physicochemical changes affect the bioavailability, rate of absorption and duration of action of different formulations of CoQ10 through ocular tissues. The greater incorporation of CoQ10 into negatively charged liposomes can explain its higher efficacy in ameliorating cataract than other liposome formulations. Although pure CoQ10 eye drops show relatively higher results in terms of the antioxidant capacity and soluble proteins, the sparse water solubility of CoQ10 enforced the use of dilute ethyl alcohol as a solvent,^9^ thus It is not a favorable choice to use in humans. Therefore, liposomal preparations still have the benefit of safe and rapid accessibility of the drug through tissues,^19^ and still retain a satisfying therapeutic efficacy.

## Conclusion

CoQ10, when incorporated in lipid bilayers, interacts actively with lipids and induces changes in their physico-chemical properties. In addition, a possible location of CoQ10 in the interfacial region of the membrane has been proposed. The present data clarify, to a certain extent, the molecular interactions of CoQ10 with membrane systems and may additionally contribute to a better understanding of CoQ10 physiological properties and the development of therapeutically advanced systems.

## Ethical Issues

The experiment was performed in accordance with the ARVO rules for use of animals in ophthalmic and vision research.

## Conflict of Interest

The author reports no conflicts of interest.
